# The lingering effects of perceived daily negative workplace gossip on next-day work alienation in China: a dual-pathway perspective of rumination and emotion

**DOI:** 10.3389/fpsyg.2026.1824972

**Published:** 2026-08-03

**Authors:** Waiseng Lou, Tingya He, Guiquan Li

**Affiliations:** 1 School of Psychological and Cognitive Sciences, Peking University, Beijing, China; 2 Key Laboratory of Machine Perception (Ministry of Education), Peking University, Beijing, China; 3 Beijing Key Laboratory of Brain-Computer Interface and Mental Health Modulation, Peking University, Beijing, China

**Keywords:** affective rumination, discrete emotion, lingering effect, perceived negative workplace gossip, problem-solving pondering

## Abstract

**Introduction:**

Existing research has predominantly confined its scope to the immediate, detrimental consequences of daily negative workplace gossip, largely overlooking its lingering effects. To address this critical limitation, we present a dual-pathway model grounded in the cognitive theory of rumination. By conceptualizing negative gossip as a signal of interpersonal “goal failure,” we suggest its effects are more nuanced and enduring than previously assumed.

**Methods:**

We examined how negative gossip triggers distinct overnight responses through a multi-method approach. Study 1 employed an online recall experiment with 132 employees to establish the causal effect of negative gossip on two distinct forms of post-work information processing: problem-solving pondering and affective rumination. Building upon this, Study 2 utilized a daily diary design with 139 employees over 9 days to dynamically examine the lagged effects of these cognitive processes.

**Results:**

Study 1 demonstrated that perceived negative gossip causes employees to engage in both problem-solving pondering and affective rumination. Multilevel analyses in Study 2 revealed that these lingering effects unfold in opposing directions: problem-solving pondering at night fosters strong emotion the next morning, which subsequently reduces work alienation. Conversely, affective rumination precipitates distress emotion the next morning, thereby exacerbating work alienation.

**Discussion:**

These findings offer critical insights into the enduring and dual-edged nature of workplace gossip. By identifying the sequential cognitive-affective chains during and after non-work time, this study expands theoretical understandings of how daily gossip shapes next-day work experiences through both maladaptive strain and constructive mobilization.

## 1 Introduction

Gossip is generally characterized as informal and evaluative communication among individuals regarding a third party who is not present (Xu L. et al., 2026; Kurland and Pelled, 2000). It serves as a core component of everyday social life (Dunbar, 2004; Emler, 1994; Methot et al., 2026). Similarly, within the organizational context, gossip is an inherent social behavior in which nearly every employee participates (Noon and Delbridge, 1993; Zhong et al., 2025b). Depending on the content of the information, gossip is broadly categorized into positive and negative forms (Zhu et al., 2022). However, drawing on the premise that negative events tend to have a more profound and significant impact than positive ones (Baumeister et al., 2001; Paolini et al., 2024), scholars have directed particular attention toward negative workplace gossip.

Existing literature predominantly highlights the detrimental consequences of negative workplace gossip for its targets. There is a strong consensus that being the subject of such gossip undermines employees’ interpersonal status, competitiveness, and workplace trust (Cheng et al., 2020; Kim et al., 2019; Yao et al., 2020; Xu L. et al., 2026). Beyond these cognitive and relational damages, targets also suffer from negative emotional experiences, including diminished self-esteem and moral disgust (Kong, 2018; Zhong et al., 2025a), which subsequently suppress positive work behaviors such as innovation and organizational citizenship (Kong, 2018; Wu et al., 2018; Xu L. et al., 2026; Zhou et al., 2019). However, recent studies have begun to challenge this strictly negative view by exploring potential functional outcomes. For instance, evidence suggests that negative gossip can paradoxically trigger adaptive responses, such as increased feedback-seeking behavior intended to repair one’s image (Zhu et al., 2023), or mitigate the detrimental effect of abusive supervision (Zhong et al., 2025b).

While previous findings have offered valuable insights into the consequences of negative gossip, a critical limitation remains. The prevailing approach typically restricts the examination of negative gossip and its outcomes to a static timeframe (e.g., Babalola et al., 2019; Wu et al., 2018; Zong et al., 2024). Furthermore, even studies adopting a daily perspective have primarily focused on how daily negative gossip influences same-day work outcomes such as work engagement (Zhong et al., 2024), largely overlooking its lagged effects, especially on the next-day work experience. This examination is critical: because perceived negative workplace gossip signals a significant “goal failure” regarding the maintenance of positive workplace relationships and reputation (Martin and Tesser, 1996; Xu L. et al., 2026), its effects are not simply dismissed when office hours end. Instead, as suggested by cognitive theories of rumination, such goal failures trigger individuals to engage in rumination during non-work time—a “class of conscious thoughts concerning one’s goals … that recur in the absence of immediate environmental demands requiring the thoughts” (Brosschot et al., 2006, p. 114). Consequently, these persistent cognitive processes highly influence the employee’s mood and behavior the following day (Foulk et al., 2026; Sonnentag and Fritz, 2015; Wang et al., 2013).

Building on this theoretical foundation, we propose that beyond immediate interpersonal damages, daily negative gossip causes a lingering impact on employees’ work alienation the next day—a negative state characterized by psychological detachment and a loss of meaning in one’s work (Hirschfeld and Feild, 2000; Kanungo, 1979; Kozhina and Vinokurov, 2020; Usman et al., 2025). First, as suggested by cognitive theories of ruminations (Martin and Tesser, 1996; Zhao L. et al., 2025), the acute discrepancy between an employee’s desired social standing and the current interpersonal threat embedded in the perception of daily negative gossip triggers cognitive processing that bifurcates into two distinct forms of rumination during the evening: affective rumination and problem-solving pondering (Cropley et al., 2012; Xu A. J. et al., 2026; Zhao L. et al., 2025). Affective rumination is defined as a cognitive state characterized by intrusive, pervasive, and recurrent thoughts about work that are negative in affective terms. In contrast, problem-solving pondering describes the type of thinking where individuals reflect on work-related issues to find solutions or re-evaluate their past actions. Both forms involve repetitive thinking about the negative gossip, impeding employees’ ability to detach from work during non-work time (Cropley et al., 2012; Xu A. J. et al., 2026; Zhao L. et al., 2025).

**FIGURE 1:**
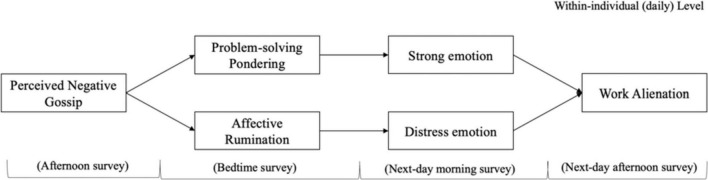
Theoretical model. Source: created by authors.

Further, we propose that these two forms of rumination differentially impact employees’ emotional states the next morning by eliciting specific discrete emotions. Specifically, affective rumination, as a maladaptive process that sustains physiological arousal and prevents psychological recovery, precipitates the discrete emotion of distress—a negative emotion characterized by significant mental strain, suffering, and emotional pain (Russell, 1980; LaRowe et al., 2024). Conversely, problem-solving pondering allows employees to cognitively resolve the “goal failure,” thereby generating the discrete emotion of strong—a positive emotion characterized by a sense of capability, determination, and vigor (Russell, 1980; LaRowe et al., 2024). Driven by these divergent discrete emotional states, employees will subsequently exhibit distinct levels of work alienation. Our theoretical model is shown in Figure 1.

Our study makes three key theoretical contributions. First, we transcend the temporal limitations of prior research by establishing a “work-home-work” spillover mechanism that extends beyond immediate effects. By identifying rumination as the critical link, we reveal how the impact of negative gossip persists during off-job time and spills back into the workplace the following day. Second, drawing on cognitive theories of rumination, we conceptualize perceived negative gossip as a form of interpersonal “goal failure,” revealing the duality of goal-coping strategies. This perspective challenges the singular narrative of victimhood, demonstrating that negative events can paradoxically stimulate constructive problem-solving efforts. Finally, we unpack the “black box” between rumination and work alienation by introducing discrete emotions as mediators. By distinguishing the specific affective pathways—whereby problem-solving pondering fosters strong emotion while affective rumination precipitates distress emotion—we provide a granular mechanism explaining how divergent cognitive processes translate into distinct workplace attitudes.

## 2 Theory and hypotheses

### 2.1 A goal-failure view of daily perceived negative gossip and rumination

As inherently social creatures (Klein et al., 2006; Baumeister, 2005; Huang et al., 2026), employees rely on positive interpersonal relationships as essential foundations for organizational adaptation, teamwork, and effectiveness (Ferris et al., 2007; Gürlek, 2021; Williams and Liu, 2022; Wu et al., 2024). Given that social behavior is fundamentally goal-directed—driven by motives to “get along” through cooperation and “get ahead” through active interaction (Hogan et al., 1998)—maintaining relational harmony constitutes a primary objective for employees. However, perceived negative workplace gossip, defined as the perception of negative evaluations and information dissemination by others (Cheng et al., 2023; Chandra and Robinson, 2010), stands as a critical obstruction to this fundamental goal. Unlike general workplace stressors, negative gossip specifically attacks an individual’s reputation and social standing, sending a potent signal of social exclusion and relational devaluation. Gossip in fact dismantles the target’s ability to maintain the social connections required for survival and success (Chandra and Robinson, 2010; Xu L. et al., 2026). Consequently, employees do not merely process negative gossip as unpleasant information, but acutely perceive it as a salient signal of goal failure—an indication that their core objective of maintaining functional workplace relationships is failing or under imminent threat.

According to cognitive theories of rumination, the perception of such goal failure serves as the primary catalyst for rumination (Martin and Tesser, 1996; Zhao L. et al., 2025). Specifically, rumination is formally defined as “conscious thoughts that revolve around a common instrumental theme and that recur in the absence of immediate environmental demands requiring the thoughts” (Martin and Tesser, 1996, p.7). Theoretical models posit that this repetitive thinking arises inevitably when an individual detects a discrepancy between their current state and a desired goal, persisting until the discrepancy is resolved or the goal is abandoned (Martin and Tesser, 1996; Nolen-Hoeksema et al., 2008; Zhao L. et al., 2025). In the workplace, human thought remains fundamentally goal-directed (Brandstätter and Bernecker, 2022; Koole et al., 1999). When employees perceive negative gossip, they are confronted with a direct violation of their goal to maintain a harmonious interpersonal environment and friendly interactions. Consequently, this discrepancy triggers employees to turn their attention inward, reflecting on and suspecting their own failures in social domains (Wang et al., 2013; Zhao L. et al., 2025). Therefore, drawing on these cognitive theories, we propose that perceived negative workplace gossip acts as a profound source that will trigger employees to engage in rumination.

Cognitive theories of rumination explicitly distinguish between two forms of post-work processing: problem-solving pondering and affective rumination (Cropley and Zijlstra, 2011; Xu A. J. et al., 2026; Zhao L. et al., 2025). Problem-solving pondering is described as a form of long-term thinking directed at a specific problem or previous work event to find a solution or improvement, without the involvement of negative affective processes. It is inherently a problem-centered response, representing a constructive attempt to develop a plan to cognitively accomplish tasks (Xu A. J. et al., 2026; Zhao L. et al., 2025). In contrast, affective rumination is defined as a cognitive state characterized by the recurrence of intrusive, negative thoughts about work and associated distress emotion. Unlike the constructive nature of problem-solving pondering, affective rumination reflects a failure to relax and contributes to increased psychological and physiological arousal (Xu A. J. et al., 2026; Zhao L. et al., 2025).

We propose that perceived negative workplace gossip—as a salient signal of interpersonal “goal failure”—will trigger both forms of rumination simultaneously. On one hand, the discrepancy between the desired state of relational harmony and the current social threat motivates individuals to consider how best to move toward an ideal outcome (Carver, 2006; Pauli et al., 2023). To dispel the negative effects of gossip and change the threatening situation, employees are likely to engage in problem-solving pondering as an adaptive effort to cognitively resolve the relational crisis (Watkins, 2008; Zhao L. et al., 2025). On the other hand, the acute threat of social devaluation makes it difficult for employees to detach during leisure time. Consistent with cognitive theories, the tension created by this goal thwarting manifests as invasive, repetitive thoughts about the gossip, leading employees to involuntarily engage in affective rumination. Thus, negative gossip acts as a dual trigger, demanding cognitive resolution while simultaneously inflicting distress emotion.

Taken together, we hypothesize that:

*Hypothesis 1a: Perceived negative gossip is positively related to problem-solving pondering.*

*Hypothesis 1b: Perceived negative gossip is positively related to affective rumination.*

### 2.2 Rumination and emotion

While perceived negative gossip signifies a failure in the pursuit of interpersonal goals, the broader literature on goal pursuit has often relegated emotions to a secondary role, primarily focusing on the general satisfaction derived from goal attainment (Plemmons and Weiss, 2013; Wax et al., 2022). Moreover, goal-setting research typically adopts a coarse-grained approach to investigating affect, relying on broad aggregates of positive and negative emotions rather than distinguishing between discrete emotional states. Critiquing this oversight, Plemmons and Weiss (2013) argue that because such broad affective summaries fail to capture the informational nuance necessary to understand distinct behavioral responses, investigating specific, discrete emotional states is essential to uncovering the divergent outcomes of goal-directed processes. Considering that the functions of the two forms of rumination diverge significantly following the perception, it is more appropriate to examine discrete emotional activation rather than general states of positive or negative affect.

To operationalize these discrete states, we adopt the circumplex model of affect, which posits that affective states are distributed in a circular space defined by two orthogonal dimensions: the degree of pleasantness and the degree of activation (Russell, 1980; LaRowe et al., 2024). Pleasantness is a subjective experience that summarizes the valence of emotional states as “positive–negative” (Seo et al., 2004). In contrast, activation refers to a sense of mobilization or energy, summarizing one’s physiological state (Seo et al., 2004; Young et al., 2024). Within this framework, we choose two discrete emotions, strong and distress, which occupy theoretical “mirror positions” (Russell, 1980; Feldman Barrett and Russell, 1998; LaRowe et al., 2024). Specifically, both states are characterized by high levels of activation—reflecting a state of high energy and arousal—yet they stand at opposite ends of the pleasantness dimension (Feldman Barrett and Russell, 1998; Young et al., 2024). As noted by Welsh et al. (2020), these emotions are natural pairings for investigation because they allow researchers to explore how a similar state of high physiological arousal can be differentiated into opposing positive and negative outcomes. Thus, distinguishing between strong and distress emotion is critical for understanding the nuanced emotional aftermath of goal-directed cognitive processes.

Based on these dimensions, we posit that problem-solving pondering is hypothesized to foster the discrete emotion of strong. Unlike passive reflection, individuals actively engage in this form of thinking to find ways to complete tasks and achieve goals, a process often driven by intrinsic interest or the drive to resolve discrepancies (Michalianou, 2011; Sun et al., 2026). Empirically, such constructive mental examination—identifying obstacles, removing barriers, and developing new perspectives—has been shown to generate positive emotions and predict next-day creativity (Weinberger et al., 2018; Xu A. J. et al., 2026). As a proactive and restorative process, problem-solving pondering provides individuals with the necessary cognitive resources (“ideas”) and psychological vitality (“strength”) to face upcoming workplace demands the following day (Bennett et al., 2016; Sun et al., 2026), which helps employees activate strong emotion—a highly activated, positive emotional state the next day, characterized by vigor and determination. According to the PANAS-X framework, strong emotion captures a distinct facet of self-assurance that diverges from the facet of joviality (e.g., feeling excited) or attentiveness (e.g., feeling alert) (Watson and Clark, 1994; Haney et al., 2023). This conceptual distinction is empirically supported by Egloff et al. (2003), who demonstrated that “strong” and “determined” consistently cluster together and exhibit unique reactivity to challenging contexts (Rominger et al., 2024). Thus, compared to other high-activated positive emotion, strong emotion more accurately captures the energetic readiness after problem-solving pondering.

Conversely, as a maladaptive and draining process, affective rumination locks individuals into a negative cognitive state that fixates on the adverse aspects of work experiences (Cropley et al., 2012; Xu A. J. et al., 2026; Zhao L. et al., 2025). This maladaptive processing does not merely reflect negative valence; it actively sustains high levels of psychophysiological arousal, preventing the return to baseline levels (Cropley et al., 2015; Janurek et al., 2024). It precipitates distress emotion—a highly activated, negative emotional state the next day, characterized by significant mental strain, suffering, and emotional pain (Russell, 1980; LaRowe et al., 2024). Compared to general negative affect, distress emotion more accurately captures the acute psychological tension and high-arousal suffering employees endure before backing workplace. By intensifying negative emotional experiences and preventing detachment, affective rumination prolongs the physiological stress response (Denson et al., 2009; Nolen-Hoeksema et al., 2008; Xu A. J. et al., 2026; Janurek et al., 2024), thereby manifesting as distress emotion the following morning.

Therefore, we hypothesize that:

*Hypothesis 2a: Problem-solving pondering is positively related to strong emotion.*

*Hypothesis 2b: Affective rumination is positively related to distress emotion.*

Building on the goal-failure framework, we propose that the specific form of ruminative processing serves as a pivotal mechanism determining whether the interpersonal setback of negative gossip translates into an emotional state of strong or distress the following morning. Specifically, by engaging in problem-solving pondering, employees perform constructive mental simulations to repair damaged relationships (Syrek et al., 2017; Xu A. J. et al., 2026), generating a sense of agency that transforms the high arousal of the goal failure into the positive mobilization of strong emotion. In stark contrast, affective rumination entraps employees in intrusive worries without resolution (Cropley et al., 2015; Zhao L. et al., 2025), converting the stress of gossip into significant psychological strain that manifests as the discrete emotion of distress. Consequently, these two distinct forms of rumination function as divergent mediators, channeling the initial goal failure of negative gossip into opposing discrete emotional outcomes.

*Hypothesis 3a: Problem-solving pondering mediated the positive relationship between perceived negative gossip and strong emotion.*

*Hypothesis 3b: Affective rumination mediated the positive relationship between perceived negative gossip and distress emotion.*

### 2.3 Work alienation

We identify work alienation as the distal work outcome of perceived negative workplace gossip. Defined as “the extent to which a person is disengaged from the world of work” (Hirschfeld and Feild, 2000, page. 790), alienation is fundamentally rooted in the quality of an individual’s connections within the organization. This state reflects not merely a transient drop in mood, but a profound psychological withdrawal resulting from the “thwarted goal of relational belonging” (Kanungo, 1979; Zhao J. et al., 2025). In our proposed model, the discrete emotional states experienced the next morning, shaped by the distinct forms of overnight rumination, serve as the proximal determinants of this outcome. We argue that these specific emotional states embody the employee’s psychological condition following the processing of negative gossip, thereby directly dictating the extent to which they socially and psychologically detach from their work.

Specifically, we posit that these divergent discrete emotions fundamentally alter the employee’s psychological proximity to their work, thereby determining the level of work alienation. The discrete emotion of strong—emerging from the constructive agency of problem-solving pondering—serves as a critical psychological resource that buffers against alienation. When employees feel strong emotion the next morning, they perceive the prior “goal failure” not as a terminal defeat, but as a challenge to be overcome (Bennett et al., 2016; Sun et al., 2026). This sense of vigor restores perceived meaning and motivates the employee to psychologically re-engage with the workplace, directly counteracting the sense of powerlessness inherent in work alienation.

Conversely, distress emotion—precipitated by the intrusive entrapment of affective rumination—acts as a potent catalyst for work alienation. Representing a state of high-arousal suffering, distress emotion signals to the employee that the workplace remains a hostile environment (Russell, 1980; Liu et al., 2025). Overwhelmed by this persistent negative activation, employees are likely to adopt a defensive posture of psychological withdrawal to protect their remaining resources, thereby deepening their sense of estrangement and loss of meaning (Kanungo, 1979; Banai and Reisel, 2007; Liu et al., 2025).

Synthesizing our theoretical arguments, we posit that the discrete emotional states experienced the next morning serve as the direct precursors to work alienation by dictating the employee’s psychological proximity to their work. Specifically, we propose that strong emotion—reflecting a restorative capacity—will be negatively associated with work alienation by fostering re-engagement, whereas distress emotion—reflecting a defensive strain—will be positively associated with work alienation by prompting psychological withdrawal. Integrating these proximal effects with our antecedent mechanisms, we propose a comprehensive dual-pathway serial mediation model wherein perceived negative workplace gossip exerts its influence on work alienation through these sequential cognitive-affective chains.

*Hypothesis 4a: Strong emotion is negatively related to work alienation.*

*Hypothesis 4b: Distress emotion is positively related to work alienation.*

*Hypothesis 5a: Problem-solving pondering and strong emotion serially mediate the negative relationship between perceived negative gossip and work alienation.*

*Hypothesis 5b: Affective rumination and distress emotion serially mediate the positive relationship between perceived negative gossip and work alienation*.

## 3 Overview of studies

We tested our hypotheses using a multi-method approach across two complementary studies, combining experimental and field data. In Study 1, an online recall experiment conducted via Credamo with currently employed participants, we established the causal effects of perceived negative workplace gossip on problem-solving pondering and affective rumination. Building upon this causal foundation, Study 2 employed a daily experience sampling method (ESM) to test the full theoretical model in a real-world context. Specifically, this field study allowed us to examine how daily negative gossip influences evening ruminations, which subsequently shape employees’ strong and distress emotions, as well as their work alienation, the following day.

### 3.1 Study 1

#### 3.1.1 Sample and procedures

In March 2024, we conducted an online recall experiment via Credamo, a widely recognized online survey and experiment platform in China (Hao et al., 2026; Huang et al., 2026). Participants were randomly recruited from the platform’s registered user pool, with a strict screening criterion that they must be currently employed. All participants were assured anonymity and confidentiality and provided full informed consent before being randomly assigned to either the gossip or the no-gossip condition. In the gossip condition, we employed a recall method to prompt participants to recollect their most vivid experience of perceived negative workplace gossip and they read the following prompt:

“Have you ever been the subject of negative workplace gossip? Please describe a specific incident that stands out to you where you experienced negative gossip in the workplace. Describe: The specific content of this negative workplace gossip; The individuals or group likely involved in making these remarks—were they colleagues, superiors, or others? Your emotions and actions upon discovering such discussions about you behind your back; Feel free to elaborate beyond these points, please write about a workplace gossip incident you recall vividly for at least 3 min.”

Under the gossip condition, a timer interface was utilized to ensure participants wrote about their perceived negative workplace gossip for at least 3 min to aid in recalling specific details.

In the control condition, participants read the following prompt:

“Please recall your activities yesterday. Now, please answer the following questions: What time did you wake up yesterday? What did you do in the morning? When did you have lunch yesterday? What did you do in the afternoon? When did you have dinner last night? What did you do in the evening? Feel free to elaborate beyond these points; please write about your experiences throughout yesterday for at least 3 min.”

Under the non-gossip condition, a timer interface was implemented to prompt participants to write about their experiences from yesterday for at least 3 min. The purpose was to assist participants in better recalling their state of mind and feelings from the previous day.

Next, participants completed a short survey assessing the manipulation check, and demographic characteristics. In addition, participants were instructed to complete the experiment material before noon, and were informed that they would receive another questionnaire in the evening, which they were requested to fill out before bedtime. The evening survey included our measures of problem-solving pondering and affective rumination.

Initially, 160 participants completed the noon experimental recall task. In the evening, 132 of these initial participants returned to complete the follow-up survey. Therefore, our final valid sample consisted of 132 participants who successfully participated in both experiment and survey (yielding a retention rate of 82.5%).

#### 3.1.2 Measures

##### 3.1.2.1 Problem-solving pondering (dependent variable)

We adapted a three-item scale to measure problem-solving pondering from Cropley et al. (2012) to focus on the extent of rumination since participants arrived home before bedtime. This scale has also been used in previous studies (Junker et al., 2021; Xu A. J. et al., 2026). Sample items included “Today, I find solutions to gossip-related problems in my free time.” The Cronbach alpha for this scale was 0.89.

##### 3.1.2.2 Affective rumination (dependent variable)

We adapted a three-item scale to measure affective rumination from Cropley et al. (2012) to focus on the extent of rumination since participants arrived home before bedtime. This scale has also been used in previous studies (Junker et al., 2021; Xu A. J. et al., 2026). Sample items included “Today, I get tense when I think about gossip-related topics in my free time.” The Cronbach alpha for this scale was 0.89.

##### 3.1.2.3 Control Variables

Given previous findings on the effects of demographic variables on workplace gossip, we also included participants’ gender, age, education, tenure, and years at work as control variables (Babalola et al., 2019; Cheng et al., 2020; Xu L. et al., 2026).

#### 3.1.3 Results

##### 3.1.3.1 Manipulation check

We adopted a three-item scale developed by Chandra and Robinson’s (2010) to assesse the efficacy of the gossip scenario, which has also been used in previous study (Xu L. et al., 2026). Sample items included “Others (e.g., co-workers and/or supervisors) communicated damaging information about me at work.” The analysis showed that participants in the gossip condition reported higher levels of gossip (*M* = 4.05, SD = 0.73) than those in the control condition (*M* = 1.88, SD = 0.77; t(130) = −16.64, *p* < 0.001).

##### 3.1.3.2 Hypothesis testing

ANOVA results showed that participants in the gossip condition reported significantly higher levels of problem-solving pondering (*M* = 3.71, *SD* = 0.85) than those in the control condition (*M* = 2.17, *SD* = 0.93, *F*(1,130) = 99.39, *p* < 0.001, *η^2^* = 0.43) and higher levels of affective rumination (*M* = 3.79, *SD* = 1.04) than those in the control condition (*M* = 2.37, *SD* = 1.04, *F*(1,130) = 61.73, *p* < 0.001, *η^2^* = 0.32). ANCOVA results, in which we entered the control variables as covariates, also indicated a significant effect of the gossip condition on problem-solving pondering (*F*(1,129) = 16.74, *p* < 0.001, *η^2^* = 0.45) and affective rumination (*F*(1,129) = 11.66, *p* < 0.001, *η^2^* = 0.36). Thus, Hypothesis 1a and 1b was supported.

### 3.2 Study 2

#### 3.2.1 Sample and procedures

To build upon Study 1’s causal findings and capture the real-world temporal sequence of our full theoretical model, Study 2 employs an experience sampling method (ESM) to dynamically examine how ruminations shape next-day discrete emotions and work alienation. We conducted a daily diary study using a snowball sampling approach in December, 2023. Initially, the authors released recruitment advertisements on popular Chinese social media platforms, including WeChat and Weibo. To be eligible, participants had to be currently employed full-time. Meanwhile, these initial participants were encouraged to simultaneously invite their colleagues to participate in the study to expand the sample network. A total of 145 people fully opted in.

The variables in this study were collected at three points during each day and in a baseline survey. The afternoon survey (sent at 17:00 and included in the data if participants responded within 2 h; average response time: 17:35) included our measures of daily perceived negative gossip and work alienation, except that the first-day survey did not assess work alienation. The evening survey (sent at 9 p.m. and included in the data if participants responded within 6 h, thus allowing participants to respond before bed regardless of the specific time; average response time: 21:32) included our measures of problem-solving pondering and affective rumination. Our morning survey (sent at 7:00 am and included in the data if participants responded within 2 h; average response time: 08:01) included our measures of positive affect and negative affect, as well as an additional control variable (sleep quality). Data collection lasted for 9 consecutive working days (Monday to Friday in the first week and Monday to Thursday in the second week, with the final morning survey sent on the second Friday).

The final between-person sample size was 139 (due to missing data) and the within-person sample size was 1028 matched daily observations. Participants were predominantly female (66%), with a mean age of 30.00 years (*SD* = 5.81), a mean number of years in working of 7.27 years (*SD* = 6.32), and a mean number of years in the job of 3.88 years (*SD* = 4.82).

#### 3.2.2 Measures

The study used the common back-translation procedure to translate all scales from English into Chinese (Brislin, 1970). Responses for all measures were given on a 5-point scale ranging from 1 = strongly disagree to 5 = strongly agree.

##### 3.2.2.1 Perceived negative workplace gossip (afternoon, t)

We adopted a three-item scale developed by Chandra and Robinson’s (2010) to measure daily perceived negative workplace gossip, which has also been used in previous study (Xu L. et al., 2026). Sample items included “Today, others (e.g., co-workers and/or supervisors) communicated damaging information about me at work.” This scale has also been used in the Chinese context in previous studies (Cheng et al., 2020; Ye et al., 2019). The Cronbach alpha for this scale was 0.94.

##### 3.2.2.2 Problem-solving pondering (evening, t)

We adapted a three-item scale to measure problem-solving pondering from Cropley et al. (2012) to focus on the extent of rumination since participants arrived home before bedtime, which was also used in Study 1. This scale has also been used in previous studies (Junker et al., 2021; Xu A. J. et al., 2026). Sample items included “Today, I find solutions to gossip-related problems in my free time.” The Cronbach alpha for this scale was 0.88.

##### 3.2.2.3 Affective rumination (evening, t)

We adapted a three-item scale to measure affective rumination from Cropley et al. (2012) to focus on the extent of rumination since participants arrived home before bedtime, which was also used in Study 1. This scale has also been used in previous studies (Junker et al., 2021; Xu A. J. et al., 2026). Sample items included “Today, I get tense when I think about gossip-related topics in my free time.” The Cronbach alpha for this scale was 0.89.

##### 3.2.2.4 Strong emotion and distress emotion (morning, t+1)

We measure strong and distress emotion using a single-item measure. This approach aligns with evidence that single items can effectively capture discrete emotions while avoiding conceptual overlap with related but distinct affective experiences (Gabriel et al., 2019), widely employed in ESM studies (Poerio et al., 2016; Prayag et al., 2020; Camerman et al., 2024). The items are “I feel strong right now” and “I feel distressed right now.”

##### 3.2.2.5 Work alienation (afternoon, t+1)

We used an eight-item scale developed by Nair and Vohra (2010) to measure work alienation. This scale has also been used in previous studies (Usman et al., 2025). Sample items included “Today I don’t enjoy my work.” The Cronbach’s alpha for this scale was 0.95.

##### 3.2.2.6 Control variables

Given previous findings on the effects of demographic variables on workplace gossip, we also included participants’ gender, age, education, tenure, and years at work as control variables, as Study 1 did. We also controlled for participants’ sleep quality to rule out an alternative explanation for the next day’s results, as previous research shows an association with evening rumination and sleep (Janurek et al., 2024; Querstret and Cropley, 2012; Syrek et al., 2017). Finally, we controlled for the previous day’s work alienation in the study.

#### 3.2.3 Analytic approach

Given the nested nature of our data, we ran a multilevel path analysis in Mplus 8 (Muthén and Muthén, 1998–2015; Speyer et al., 2024). We first partitioned the variance in our level 1 constructs to confirm the within-person variability. The results (see Table 1) showed that the variance percentages within persons ranged from 34% to 70%, supporting multilevel analyses. More importantly, 65% of the variance in ratings of daily perceived negative gossip was within-person, supporting our within-person focus.

**TABLE 1:** Variance decomposition for within-person variables.

Variable	Within-personvariance(σ^2^)	Between-person variance (τ _oo_)	Percentage of total variancewithin-person
Daily perceived negative gossip (afternoon)	0.30	0.16	65%
Work alienation (afternoon)	0.30	0.49	38%
Problem-solving pondering (evening)	0.58	0.47	55%
Affective rumination (evening)	0.50	0.41	55%
Sleep quality (morning, t+1)	0.53	0.23	70%
Strong emotion (morning, t+1)	0.62	0.43	59%
Distress emotion (morning, t+1)	0.65	0.33	66%
Work alienation (afternoon, t+1)	0.28	0.55	34%

Percentage of total variance within-person was calculated as the following: σ^2^/(σ^2^+ τ_*o*__*o*_). Estimates are based on the total Level-1 sample size before testing the lagged effects (*n* = 1028). Within-person variance decomposition for the lagged variables is available upon request.

Before running our analyses, we first conducted confirmatory factor analyses (CFAs) to test the discriminant validity of the multi-item variables in the study. The hypothesized six-factor model (χ^2^ (196) = 1013.80, CFI = 0.91, TLI = 0.90, SRMR = 0.04, RMSEA = 0.06) fit the data better than an alternative model, including a five-factor model in which problem-solving pondering and affective rumination were combined into one factor (χ^2^ (201) = 1486.87, CFI = 0.85, TLI = 0.83, SRMR = 0.06, RMSEA = 0.08); a 4-factor model in which problem-solving pondering and affective rumination were combined into one factor and strong emotion and distress emotion into the other (χ^2^ (203) = 1627.08, CFI = 0.85, TLI = 0.83, SRMR = 0.07, RMSEA = 0.08); a 3-factor model in which problem-solving pondering, affective rumination, strong emotion, and distress emotion were combined into one factor (χ^2^ (206) = 1814.71, CFI = 0.83, TLI = 0.81, SRMR = 0.11, RMSEA = 0.09); a 2-factor model in which perceived negative gossip, problem-solving pondering, affective rumination, strong emotion, and distress emotion were combined into one factor (χ^2^ (208) = 3411.417, CFI = 0.65, TLI = 0.62, SRMR = 0.16, RMSEA = 0.12); and a one-factor model in which all items loaded on one factor (χ^2^ (231) = 9490.58, CFI = 0.41, TLI = 0.35, SRMR = 0.21, RMSEA = 0.16).

#### 3.2.4 Results

Table 2 shows the means, standard deviations and bivariate correlations. Given that the purpose of our study is to examine the daily impact of perceived negative workplace gossip on employees’ work alienation, the coefficients for our hypothesized relationships are all reported at the within-individual level. The results of multilevel path analysis are presented in Table 3. In support of hypothesis 1a and 1b, daily perceived negative gossip was positively related to (a) problem-solving pondering (γ = 0.26, *p* < 0.01) and (b) affective rumination (γ = 0.30, *p* < 0.001). Problem-solving pondering was positively related to the next day’s strong emotion (γ = 0.23, *p* < 0.001) and affective rumination was positively related to the next day’s distress emotion (γ = 0.17, *p* < 0.01), meaning that hypothesis 2a and 2b was supported. Supporting Hypotheses 3a and 3b, the indirect effect of daily perceived negative gossip via problem-solving pondering on strong emotion (estimate = 0.06; 95%CI = [0.02, 0.10]) and daily perceived negative gossip via affective rumination on distress emotion (estimate = 0.05; 95%CI = [0.02, 0.10]). Strong emotion was negatively related to work alienation (γ = −0.10, *p* < 0.001) and distress emotion was positively related to work alienation (γ = 0.08, *p* < 0.01), supporting Hypothesis 4a and 4b. Finally, Hypothesis 5a and 5b, respectively, addressed the serial indirect effect of daily perceived negative workplace gossip on work alienation via (a) problem-solving pondering and strong emotion (indirect effect = −0.01, 95%CI = [−0.01, −0.00]) and (b) affective rumination and distress emotion (indirect effect = 0.00, 95%CI = [0.00, 0.01]). The serial indirect effect was positive and significant, supporting hypothesis 5a and 5b.

**TABLE 2:** Means, standard deviations, and correlations between variables.

Variable	M	Within-person*SD*	Between-person*SD*													
				1	2	3	4	5	6	7	8	9	10	11	12	13
1. Gender	0.34		0.47	–	–	–	–	–								
2. Age	30.00	5.81	0.05	-												
3. Education	3.17	0.72	−0.17**	−0.30**	–											
4. Years of working	7.27	6.32	0.03	0.94**	−0.38**	–										
5. Job Tenure	3.88	4.82	0.01	0.69**	−0.12**	0.69**	–									
6. Perceived negative gossip (afternoon)	2.40	0.68	0.46	0.04	0.10**	−0.19**	0.13**	0.08**	(0.94)	0.39**	0.15**	0.26**	−0.07*	0.19**	−0.03	0.30**
7. Work alienation (afternoon)	2.68	0.90	0.73	−0.02	−0.07*	0.01	−0.06	−0.01	0.39**	(0.95)	−0.03	0.19**	−0.32**	0.39**	−0.13**	0.72**
8. Problem-solving pondering (evening)	2.75	1.02	0.75	0.03	0.07*	−0.13**	0.08*	0.00	0.15**	−0.03	(0.88)	0.60**	0.24**	0.08*	0.08*	−0.09**
9. Affective rumination (evening)	2.53	0.95	0.70	−0.00	0.07*	−0.06*	0.06	0.07*	0.26**	0.19**	0.60**	(0.89)	0.03	0.24**	−0.06	0.12**
10. Strong emotion (next morning)	3.17	1.03	0.72	0.07*	0.09**	−0.08*	0.08*	−0.05	−0.07*	−0.33**	0.24**	0.03	-	−0.37**	0.29**	−0.38**
11. Distress emotion (next morning)	2.40	1.09	0.69	0.02	−0.05	−0.01	−0.05	−0.04	0.19**	0.39**	0.08*	0.24**	−0.37**	-	−0.24**	0.40**
12. Sleep quality (next morning)	3.28	0.87	0.58	0.07*	0.03	−0.08**	0.07*	0.02	−0.03	−0.13**	0.08*	−0.06	0.29**	−0.24**	-	−0.18**
13. Work alienation (next afternoon)	2.69	0.91	0.78	−0.01	−0.07*	0.02	−0.06	−0.03	0.30**	0.72**	−0.09**	0.12**	−0.38**	0.40**	−0.18**	(0.95)

Correlations below the diagonal represent between-person correlations (*N* = 139). Within-person variables were averaged across days to form the between-person variables. Correlations above the diagonal represent within-person correlations (*N* = 1,028). Coefficient alpha estimates of reliability are in parentheses on the diagonal. For within-person variables, their reliabilities were the mean alphas across 8 days of observation. Gender (0 = woman, 1 = man). Education (1 = Senior secondary education 2 = Junior college education 3 = Undergraduate education 4 = Graduate education). *p < 0.05, **p < 0.01.

**TABLE 3:** Simultaneous multilevel path analysis results for hypothesized model.

	Problem-solving pondering(evening, t)		Affective rumination(evening, t)		Strong emotion(morning, t+1)		Distress emotion(morning, t+1)		Work alienation(afternoon, t+1)	
Predictor	γ	*SE*	γ	*SE*	γ	*SE*	γ	*SE*	γ	*SE*
Gender	0.01	(0.13)	0.03	(0.12)	−0.08	(0.10)	−0.08	(0.08)	−0.01	(0.05)
Age	0.02	(0.03)	0.03	(0.03)	0.04	(0.03)	−0.02	(0.02)	0.00	(0.01)
Education	−0.12	(0.10)	−0.05	(0.08)	0.00	(0.07)	−0.02	(0.06)	−0.01	(0.03)
Years of working	−0.01	(0.03)	−0.03	(0.02)	−0.01	(0.03)	0.01	(0.02)	0.00	(0.01)
Job tenure	−0.02	(0.02)	0.01	(0.01)	−0.03	(0.01)	−0.01	(0.01)	−0.00	(0.01)
Daily perceived negative gossip (afternoon)	0.26**	(0.08)	0.30***	(0.07)	0.03	(0.06)	0.02	(0.06)	0.06	(0.05)
Work Alienation (afternoon)	−0.10	(0.07)	0.11^+^	(0.06)	−0.31***	(0.06)	0.36***	(0.05)	0.64***	(0.04)
Problem-solving pondering (evening)					0.23***	(0.05)	0.00	(0.05)	−0.05*	(0.03)
Affective rumination (evening)					−0.06	(0.05)	0.17**	(0.05)	0.01	(0.03)
Sleep quality (next morning)					0.28***	(0.05)	−0.22***	(0.04)	−0.04	(0.03)
Strong emotion (next morning)									−0.10***	(0.03)
Distress emotion (next morning)									0.08**	(0.03)

*N*_*level–1*_ = 1028 (accounted for data lagged to the next workday). *N*_*level–2*_ = 139. ^+^*p* < 0.10, *p < 0.05, **p < 0.01, ***p < 0.001.

## 4 Discussion

Using a multi-method approach, our research extends the burgeoning literature on negative workplace gossip by illuminating the continuous, day-to-day dynamic mechanisms that link perceived gossip to work alienation. In Study 1, we first establish robust causal evidence that perceived negative workplace gossip acts as a “goal-failure” signal simultaneously triggering both problem-solving pondering and affective rumination. This experimental approach explicitly extends previous research that has predominantly relied on correlational designs to examine the relationship between negative gossip and cognitive constructs (Cheng et al., 2023; Xu L. et al., 2026). Building upon this causal foundation, Study 2 utilizes a daily experience sampling method to unpack the unfolding temporal sequence. By capturing within-person, cross-day fluctuations, we demonstrate that distinct overnight ruminations triggered by negative gossip differentially shape employees’ next-day psychological states: problem-solving pondering fosters strong emotion the next morning to buffer against work alienation, whereas affective rumination precipitates distress emotion to exacerbate it. By illuminating this daily dynamic chain, our findings offer a more nuanced understanding of gossip’s lingering effects, explicitly moving beyond traditional literature that relies on static cross-sectional designs, broad time-lagged intervals, or strictly same-day outcomes (Cheng et al., 2023; Xu L. et al., 2026; Zhong et al., 2025b).

### 4.1 Theoretical implications

First, we contribute to the literature by establishing a work-home-work spillover mechanism that transcends the temporal boundaries of previous studies. While recent research of gossip often halts at the level of same-day workplace dynamics (Zhong et al., 2024; Kong, 2018; Outlaw and Baer, 2024), we apply cognitive theories of rumination to posit that perceived negative gossip implies a goal-failure event, prompting individuals to ruminate during off-job time. This process signifies the emergence of spillover effects, where rumination serves as the critical link between same-day gossip and next-day work attitudes. Empirically, we show that this spillover effect of perceived negative gossip manifests through two distinct paths: an affective rumination pathway that exacerbates work alienation via distress emotion, and a problem-solving pondering pathway that reduces alienation via strong emotion. By explicating how negative gossip permeates non-work cognition before spilling back into the workplace to shape future alienation, our study verifies a comprehensive image of influence and broadens the theoretical horizon of gossip research.

Second, drawing on cognitive theories of rumination, we conceptualize perceived negative gossip as a “goal failure,” revealing that such failure can drive employees to proactively strive to repair the damage rather than merely being passively harmed. Prior research, often grounded in Conservation of Resources (COR) or Affective Events Theory (AET), has predominantly highlighted the detrimental effects of negative gossip (Babalola et al., 2019; Cheng et al., 2023); however, when individuals regard perceived negative workplace gossip as a goal failure, they are compelled to engage in a self-repair process to cope with (Zhu et al., 2023). Our study finds that while perceived negative gossip indeed traps individuals in affective rumination during non-work hours, it simultaneously triggers problem-solving pondering. Specifically, while the former exacerbates negative interpersonal states such as work alienation, problem-solving pondering enables individuals to regain confidence (i.e., strong emotion), thereby reducing their sense of alienation. This finding provides a more dialectical perspective on the consequences of gossip (Omanović and Langley, 2023), demonstrating that “bad” events can paradoxically stimulate constructive cognitive efforts alongside negative emotional processing.

Third, we contribute to the literature by unpacking the “black box” between ruminations and workplace behavior. Although extensive research has established the link between daily rumination and next-day work outcomes (e.g., Foulk et al., 2026; Gabriel et al., 2019; Liao et al., 2021), these studies often overlook the underlying micro-mechanisms—specifically, how the lingering effects of rumination traverse the time lag of sleep to impact the subsequent workday. Our findings illuminate two distinct affective pathways active the next morning: problem-solving pondering instills strong emotion, which effectively buffers against work alienation; conversely, affective rumination precipitates distress emotion, which significantly exacerbates such alienation feeling at work. By identifying these discrete emotional mediators, we provide a novel but critical mechanism explaining how specific forms of rumination translate into distinct workplace attitudes, thereby expanding the theoretical boundaries of rumination research.

### 4.2 Practical implications

The findings of this study offer several significant implications. First, managers should adopt a dialectical perspective toward negative workplace gossip (Omanović and Langley, 2023). While prior studies have established that negative gossip is widespread and typically exerts detrimental effects (Babalola et al., 2019; Kong, 2018; Wu et al., 2018; Xu L. et al., 2026; Ye et al., 2019), our research provides a novel insight: even when faced with such social adversity, the final outcomes heavily depend on employees’ cognitive processing styles. Therefore, managers should focus on guiding employees to view the situation from a problem-solving perspective rather than interpreting it solely as a threat. However, as Zhong et al. (2025b) suggested, we are by no means encouraging workplace negative gossip given its well-documented adverse effects. Instead, organizations should proactively implement specific upstream strategies, such as establishing formal, legitimate grievance channels. By doing so, employees have constructive avenues to express their concerns and vent their perceptions of unfair treatment, thereby preempting their reliance on destructive gossip.

Second, our findings suggest that altering the form of rumination is a critical step in preventing negative emotional spirals and reducing work alienation. Employees should be encouraged to actively adapt their thinking styles during off-job time. Research indicates that blocking negative rumination and redirecting attention to recovery activities is an effective strategy. For instance, engaging in sports or learning new hobbies can be beneficial (Li et al., 2024; Sonnentag et al., 2008). The sense of accomplishment and mastery gained from achieving goals in these non-work domains can compensate for the frustration of interpersonal goal failure at work, thereby reducing the negative states experienced by employees.

Third, organizations should refine their recruitment and training policies to foster adaptive cognitive processing. Given that affective rumination exacerbates the negative impact of gossip, companies may consider recruiting candidates who demonstrate proactive and problem-solving-oriented thinking styles during the selection process. Furthermore, since work rumination fluctuates based on work experiences, organizations should implement targeted interventions to help employees—especially those prone to affective rumination—transition toward problem-solving pondering. For example, training programs aimed at improving problem-solving skills can enhance employees’ confidence in meeting job demands, enabling them to analyze problems rationally rather than succumbing to negative emotions after work (Firoozabadi et al., 2018; Xu A. J. et al., 2026). Additionally, organizations can encourage employees to keep a narrative diary focusing on facts and behaviors. This practice promotes the cognitive reconstruction of work events, assisting employees in regulating negative rumination and gaining control over their emotional responses (Lukenda et al., 2025; Yukawa, 2008).

## 5 Limitations and future directions

Although our research findings have important theoretical and practical implications, they should be explained on the basis of some limitations. First, regarding methodology, our reliance on self-reported data raises potential concerns about common method variance (Podsakoff et al., 2003; Xu A. J. et al., 2026). Although we prioritized self-reports because our core variables—perceived gossip, internal rumination, and emotional states—are inherently phenomenological and best captured by the employees themselves, this approach may introduce bias. However, we mitigated this concern by employing a time-lagged design with data collected at four distinct time points. Furthermore, by controlling for the previous night’s sleep quality (Syrek et al., 2017; Sladek et al., 2020; Janurek et al., 2024) and the previous day’s work alienation, we rigorously isolated the daily fluctuations in our focal variables. To further strengthen causal inference and reduce measurement bias, future research could adopt multi-source designs (e.g., obtaining reports of rumination or mood from family members) or utilize objective measures (e.g., physiological records or experimental manipulations of gossip exposure).

Second, the cultural context of our study may limit the generalizability of our findings. Conducted in China, where the concept of guanxi (interpersonal connection) is paramount (Chen et al., 2013; Cheng et al., 2023), our results reflect a setting where harmonious relationships are viewed as an essential goal. Consequently, the “goal failure” triggered by gossip might be more salient here than in cultures with lower relational orientation. Future research is encouraged to replicate this model in diverse cultural contexts to examine the cross-cultural universality of these goal-regulatory mechanisms.

Third, our study focused exclusively on the victim’s perspective. While critical for understanding individual well-being, this scope overlooks the broader organizational dynamics. Future scholars could expand this line of inquiry by adopting an organizational perspective (Aljbour et al., 2025), exploring the macro-level mechanisms of how gossip operates (Vaid and Donthu, 2023), whether it serves any functional purposes for the organization, and how it can be effectively governed.

One important direction is to explore boundary conditions that influence the choice of ruminative pathways. While our model establishes rumination as a response to goal failure, individual differences may determine the type of rumination engaged. For instance, traits such as mindfulness or interpersonal sensitivity might buffer the negative effects by predisposing employees toward adaptive problem-solving pondering rather than maladaptive affective rumination (Babalola et al., 2019; Cheng et al., 2020; Zhang et al., 2026). We encourage future research to identify specific traits or contextual factors that empower employees to adopt constructive thinking patterns in the face of interpersonal setbacks.

Another avenue is to broaden the scope of outcome variables. While we focused on discrete emotions (strong and distress) as key indicators, other physiological and behavioral outcomes warrant investigation. For example, future research could examine how different forms of rumination impact sleep quality or family-related function, which in turn may influence next-day work behaviors (Syrek et al., 2017; Junker et al., 2025). Constructively replicating our model with such objective or physiological variables would further validate the practical significance of our theory.

Finally, future research could apply our “goal-based” framework to the domain of positive workplace gossip. Our study confirmed that negative gossip triggers rumination by hindering the social goal of maintaining good relationships (i.e., goal obstruction). Following this logic, positive workplace gossip might facilitate these goals (i.e., goal consistency). It would be theoretically novel to investigate whether positive gossip triggers specific forms of positive rumination through a goal-consistency mechanism (Lian et al., 2023), thereby offering a more comprehensive understanding of how gossip shapes employee cognition.

## 6 Conclusion

Grounded in the cognitive theory of rumination, this study hypothesized a dual-pathway model in which perceived negative workplace gossip triggers distinct cognitive responses, which sequentially shape employees’ next-day discrete emotions and work alienation. Our multi-method results fully corroborated all our hypotheses. Specifically, in support of Hypotheses 1a and 1b, which posited that perceived negative workplace gossip is positively related to problem-solving pondering and affective rumination, respectively, our results showed significant positive associations (γ = 0.26, *p* < 0.01; γ = 0.30, *p* < 0.001). Corroborating Hypotheses 2a and 2b, which proposed that problem-solving pondering and affective rumination are positively related to the next day’s strong emotion and distress emotion, we observed consistent positive relationships (strong emotion: γ = 0.23, *p* < 0.001; distress emotion: γ = 0.17, *p* < 0.01). Furthermore, supporting Hypotheses 3a and 3b, we found that problem-solving pondering (indirect effects = 0.06, 95%CI = [0.02, 0.10]) and affective rumination (indirect effects = 0.05; 95%CI = [0.02, 0.10]) significantly mediated the relationships between gossip and their corresponding emotions. Consistent with Hypotheses 4a and 4b, which predicted that strong emotion would negatively influence work alienation while distress emotion would positively influence it, our findings confirmed a negative effect for the former (γ = −0.10, *p* < 0.001) and a positive effect for the latter (γ = 0.08, *p* < 0.01). Finally, our serial mediation analysis supported Hypotheses 5a and 5b, demonstrating that perceived negative workplace gossip exerts significant indirect effects on work alienation via problem-solving pondering and strong emotion (indirect effects = −0.01, 95%CI = [−0.01, −0.00]), and via affective rumination and distress emotion (indirect effects = 0.00; 95%CI = [0.00, 0.01]). All assumed hypotheses got supported. We hope that our research inspires future scholars to adopt a more balanced perspective, further exploring how employees can transform the “goal failure” of negative gossip into psychological resilience and constructive work states.

## Data Availability

The raw data supporting the conclusions of this article will be made available by the authors, without undue reservation.
